# IL-6-Mediated Macrophage–Fibroblast Communication in Pathological Lung Remodeling

**DOI:** 10.3390/ijms27219979

**Published:** 2026-11-07

**Authors:** Nelya R. Chicherina, Ekaterina O. Gubernatorova, Olga A. Namakanova, Fedor A. Sysonov, Ekaterina A. Gorshkova, Marina S. Drutskaya

**Affiliations:** 1 Immunobiology and Biomedicine Division, Center for Genetics and Life Sciences, Sirius University of Science and Technology, 354340 Sochi, Russia; nelyayakhina@mail.ru; 2 Laboratory of Molecular Mechanisms of Immunity, Engelhardt Institute of Molecular Biology, Russian Academy of Sciences, 119991 Moscow, Russia; ekaterina.gubernatorova412@gmail.com (E.O.G.); olga.namakanova@gmail.com (O.A.N.); fsysonov@yandex.ru (F.A.S.); gorshsama@gmail.com (E.A.G.); 3 Belozersky Institute of Physico-Chemical Biology, Lomonosov Moscow State University, 119991 Moscow, Russia

**Keywords:** pulmonary fibrosis, SAMacs (scar-associated macrophages), myofibroblasts, senescence, inflammaging, immunometabolism, anti-IL-6 therapy

## Abstract

Fibrosis is a pathological form of tissue remodeling driven by excessive deposition of extracellular matrix (ECM). Unlike physiological tissue repair, fibrosis arises from persistent myofibroblast activation and unresolved inflammation. Chronic inflammation can both initiate fibrotic remodeling and be sustained by the resulting tissue alterations, supporting the view of fibrosis as an immune-mediated process. Fibrogenesis is fundamentally shaped by interactions between macrophages and fibroblasts. Polarized macrophages can modulate fibroblast activity and promote their differentiation into myofibroblasts. IL-6 is an important mediator of macrophage–fibroblast communication and has been implicated in fibrotic diseases affecting multiple organs. Notably, the effects of IL-6 are highly context-dependent. During acute injury, IL-6 can promote tissue repair by stimulating epithelial proliferation and angiogenesis, whereas persistent IL-6 signaling in chronic inflammation can become a major driver of fibrosis. IL-6 is also a prominent component of the senescence-associated secretory phenotype (SASP), providing a mechanistic link between fibrosis, cellular senescence, and inflammaging. In this review, we provide an integrated overview of the role of IL-6 in macrophage–fibroblast interactions during pulmonary fibrogenesis, with particular emphasis on its context-dependent functions at the interface between tissue repair and pathological tissue remodeling. We also discuss current therapeutic strategies aimed at targeting IL-6 signaling.

## 1. Introduction

Fibrosis, the replacement of normal parenchyma with connective tissue, underlies a wide range of diseases of diverse etiology and anatomical location. It may develop as a primary disorder or accompany and exacerbate pre-existing chronic conditions. Regardless of etiology, fibrotic remodeling is typically triggered by persistent parenchymal injury, which initiates a reparative response that becomes dysregulated as disease progresses [1]. Inflammation and cellular senescence are major factors that shift this balance from physiological tissue repair toward maladaptive tissue remodeling [2,3].

This review focuses on fibrosing interstitial lung diseases (fILDs), the most common of which include idiopathic pulmonary fibrosis (IPF) and connective tissue disease-associated interstitial lung disease (CTD-ILD) [4]. Survival in patients with fILD ranges from 3 to 7 years [5], while in IPF it may be reduced to approximately 4 years in the absence of lung transplantation [6]. Effective treatment options remain limited, underscoring the need for a deeper understanding of the pathogenesis of fibrosis and the molecular mechanisms that drive its progression.

**Figure 1:**
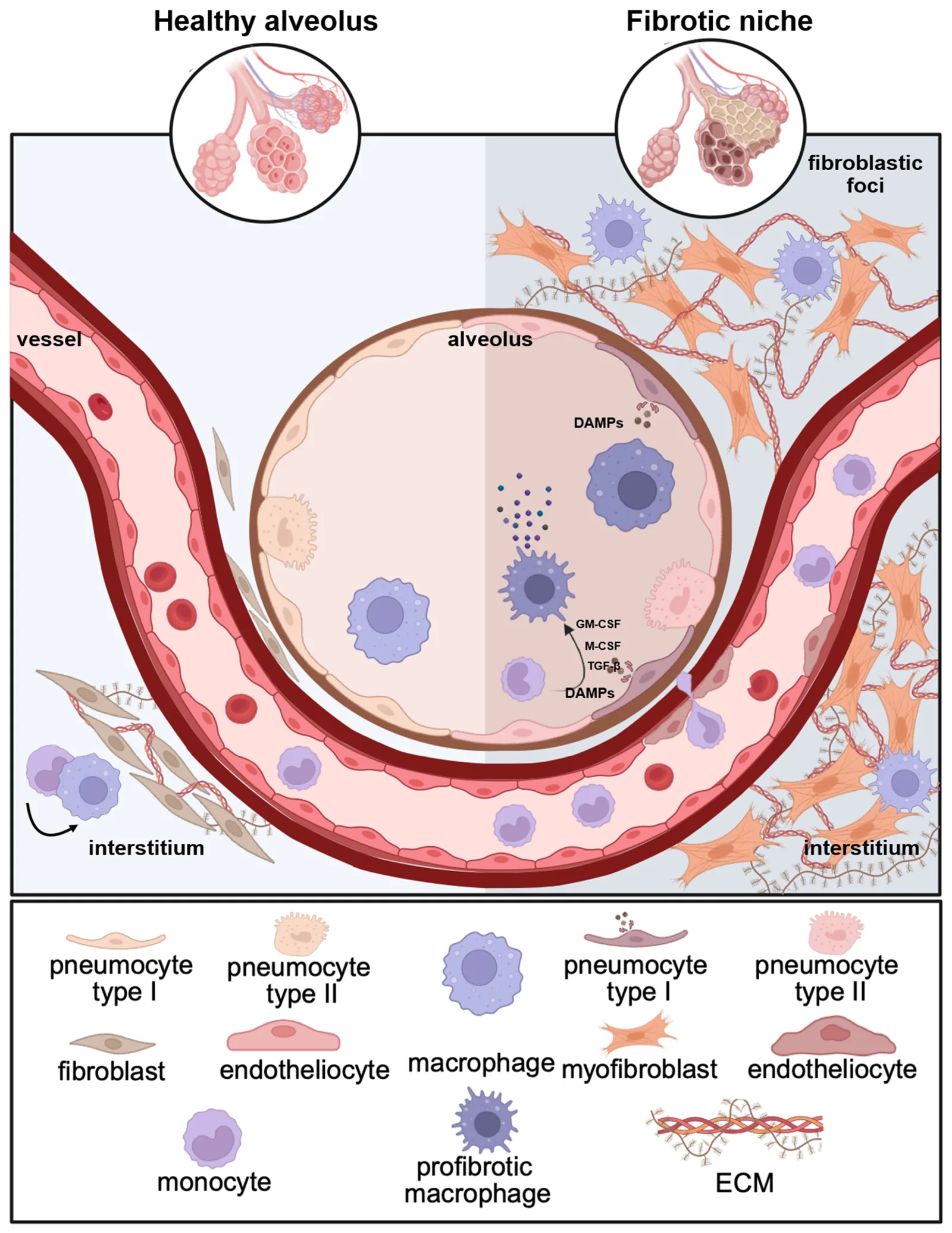
Formation of the fibrotic niche in fILD. Upon injury to the alveolar epithelium, damage-associated molecular patterns (DAMPs) are released, recruiting immune cells to the lungs. Circulating monocytes migrate into the tissue, where GM-CSF, TGF-β [13,14], and M-CSF [15] drive their differentiation into monocyte-derived alveolar macrophages, which subsequently acquire a pro-fibrotic phenotype in response to microenvironmental cues. Soluble factors secreted by pro-fibrotic macrophages recruit fibroblasts to the site of injury and trigger their differentiation into myofibroblasts, which overproduce collagen and fibronectin. The resulting accumulation of extracellular matrix components leads to replacement of the lung parenchyma with scar tissue. Crosstalk between pro-fibrotic macrophages and fibroblasts underlies the characteristic morphological features of fILD, including extracellular matrix deposition in the interstitium, formation of fibroblastic foci, thickening of the alveolar walls, and bronchiolization of the alveolar epithelium. Together, these changes drive architectural tissue reorganization and the development of honeycombing. Created in BioRender. Gubernatorova, E.O. (2026) https://BioRender.com/ts8guhp.

The characteristic morphological features of fILD, particularly IPF, include extracellular matrix accumulation within the interstitium, thickening of the alveolar walls, bronchiolization of the alveolar epithelium, formation of fibroblastic foci, and honeycombing, the ultimate catastrophic consequence of the pathological lung remodeling [7] (Figure 1). Fibrogenesis is driven by interactions between immune cells, particularly macrophages, and the stromal compartment, including myofibroblasts. Direct macrophage–myofibroblast interactions are mediated by integrin- and cadherin-based contacts [8,9]. For example, cadherin-11–based contacts retain TGF-β–producing macrophages in close proximity to fibroblasts [9]. Contact-dependent mechanisms complement soluble mediator signaling and provide spatially restricted regulation of fibroblast activation and maintenance of their pro-fibrotic phenotype. In addition to direct cell–cell interactions, soluble mediators are critical for establishing the fibrotic niche and sustaining pro-fibrotic fibroblast activation: activated macrophages and myofibroblasts secrete inflammatory mediators that promote fibrosis progression [10]. IL-6 is one such mediator produced by activated macrophages and myofibroblasts and is capable of regulating both inflammation and tissue remodeling [11,12]. This review summarizes current experimental evidence on the role of IL-6 in macrophage–fibroblast crosstalk during pulmonary fibrosis and discusses existing therapeutic approaches targeting IL-6 signaling. Importantly, the contribution of IL-6 to pulmonary fibrosis remains controversial, with experimental studies reporting both protective and pathogenic effects of IL-6 signaling. We propose that these discrepancies may reflect differences in the cellular sources of IL-6, the mode of IL-6 signaling, the timing of its activity during disease progression, and the experimental models employed. Understanding these context-dependent effects is essential for identifying the conditions under which therapeutic targeting of IL-6 may be beneficial.

## 2. Fibrosis as an Immune-Mediated Disease

Pulmonary fibrosis may develop as a consequence of recurrent injury and impaired regeneration of the alveolar epithelium [16]. Disruption of epithelial barrier integrity triggers the release of damage-associated molecular patterns (DAMPs) and pro-inflammatory mediators, initiating pathological lung remodeling [17,18]. Although inflammation is a major component of the pathogenesis of fibrosing interstitial lung diseases, including CTD-ILD, its role in IPF remains controversial [4]. Moreover, in IPF the contribution of the immune system to pathogenesis is mediated mainly by innate immunity. In contrast, in CTD-ILD the numbers of T cells and memory B cells are increased and well-formed tertiary lymphoid structures are present in the lungs, indicating active engagement of adaptive immunity [19]. Consistent with this, CTD-ILD is characterized by autoimmune-mediated organ damage, which distinguishes it from the primarily epithelial-driven pathogenesis of IPF. This distinction is reflected in therapeutic responses: immunosuppressive therapy can be effective in CTD-ILD [4], whereas it is associated with worse outcomes in patients with IPF [20]. Although pro-fibrotic remodeling can, under some conditions, proceed independently of the immune system [21], fibrosis is generally shaped by the regenerative capacity of the tissue and the nature of the accompanying immune response [22]. Fibrosis can arise as a consequence of inflammation and, in turn, sustain it [23]. The involvement of numerous cellular subsets and molecular pathways of both innate and adaptive immunity in fILD, therefore, supports the view of fibrosis as an immune-mediated pathology [5].

Different types of immune responses, including Th1-, Th2-, and Th17-associated pathways, can contribute to pulmonary fibrosis, although their effects are highly context-dependent [24]. Th1-associated responses may promote inflammation and tissue injury, whereas IFN-γ and IL-12 can exert anti-fibrotic effects, partly through antagonism to TGF-β signaling [25,26,27,28]. Th2-associated cytokines, particularly IL-4 and IL-13, promote fibroblast activation, myofibroblast differentiation, and extracellular matrix deposition [29,30,31,32]. Similarly, Th17-associated IL-17A contributes to fibrogenesis by enhancing TGF-β production and neutrophil recruitment [33,34]. Thus, distinct immune programs can converge on shared pro-fibrotic mechanisms, while their overall impact depends on the inflammatory context and stage of disease (Table 1).

## 3. Macrophages and Fibroblasts as Key Players in Fibrosis

Fibrosis is shaped by extensive crosstalk between immune cells and fibroblasts [24], with stromal cells actively shaping immune cell persistence within tissue-like niches [55]. The inflammatory milieu determines the activation and polarization state of macrophages, which are among the earliest responders to tissue injury and can critically influence the outcome of inflammation [22,28]. This places macrophages at the center of fibrotic niche formation. Mediators released by activated macrophages promote fibroblast differentiation into myofibroblasts. In turn, myofibroblasts not only respond to inflammatory signals but also actively modulate macrophage function. This bidirectional communication is mediated by soluble factors, including pro-inflammatory cytokines, TGF-β, and platelet-derived growth factor (PDGF), which promote excessive extracellular matrix production by myofibroblasts [21].

### 3.1. Pulmonary Macrophages: A Heterogeneous Population in Health and Fibrosis

Macrophages are highly heterogeneous, reflecting both their developmental origin and their capacity for reversible polarization. In the healthy human lung, two major populations are distinguished by anatomical location: alveolar macrophages, which reside within the alveolar lumen, and interstitial macrophages, which occupy the pulmonary interstitium, including the alveolar septa and peribronchial and perivascular regions [35]. Alveolar macrophages and a population of primitive interstitial macrophages located in perivascular and peripheral regions are of embryonic origin, whereas the major interstitial macrophage pool is replenished by circulating monocytes [36]. These ontogenetic differences are accompanied by functional specialization: tissue-resident macrophages contribute to tissue homeostasis and normal organ function, whereas expansion of monocyte-derived macrophages is a hallmark of inflammation [22,37]. In response to inflammatory cues, circulating monocytes migrate into tissues, where local microenvironmental signals shape their polarization and subsequent roles in inflammation and tissue repair [21,38].

Several studies have demonstrated a major contribution of monocyte-derived alveolar macrophages to the development of fibrosis [39,40]. Macrophages within the fibrotic niche, termed scar-associated macrophages (SAMacs), display a conserved transcriptional profile, suggesting the existence of a core molecular signature shared across organs [21], in contrast to the tissue-specific marker profiles of resident macrophages. scRNA-seq studies have revealed profound reshaping of the macrophage compartment in IPF [21]. To this end, in the lungs of mice with experimentally induced fibrosis, monocyte-derived alveolar macrophages with high TREM2 expression are abundant [37]. Macrophages expressing *SPP1* (secreted phosphoprotein 1) predominate in the lungs of patients with IPF and have been linked to bone marrow-derived populations based on expression of *MAFB*, *CD163*, and *LGMN* [56]. Notably, *SPP1*^+^ macrophages have also been identified in the lungs of patients with severe COVID-19 accompanied by fibrotic remodeling [57].

The pro-fibrotic properties of monocyte-derived macrophages are associated with their expression of factors involved in fibrosis and tissue repair, including *TGF-β*, *RELMα*, *PDGF*, and matrix metalloproteinases (*MMPs*) [28]. TGF-β promotes fibroblast migration, activation, and collagen synthesis [41,42,43]; RELMα supports collagen fiber crosslinking [44]; and PDGF stimulates fibroblast proliferation and chemotaxis [45]. The pro-fibrotic role of monocyte-derived macrophages is further supported by the attenuation of fibrosis following their depletion in multiple experimental models [21,39,46].

Macrophage polarization plays a context-dependent role in pulmonary fibrosis [22,28]. Pro-inflammatory M1-like macrophages contribute to early lung injury through the production of inflammatory mediators, including IL-1β, IL-6, and TNF [47,48,49,50,51]. They can also promote fibroblast recruitment and stromal activation through fibronectin and PDGF production [52,53,54]. However, M1-like macrophages may exert anti-fibrotic effects through matrix metalloproteinases and CXCL11-mediated suppression of pro-fibrotic M2-like macrophages [58,59]. Consistently, experimental reprogramming of M2-like macrophages toward an M1-like phenotype has been shown to attenuate pulmonary fibrosis [60].

In contrast, M2-like macrophages, including M2a and M2c populations identified in IPF lungs, are generally associated with fibrotic remodeling through the production of TGF-β and other pro-fibrotic mediators [47,61]. In addition to promoting fibroblast activation, M2-like macrophages can suppress anti-fibrotic programs in stromal cells, further reinforcing the fibrotic niche [62,63]. Nevertheless, the conventional M1/M2 classification does not fully capture macrophage heterogeneity in fibrotic tissues, where distinct activation states may coexist and evolve in response to local microenvironmental signals.

Thus, we define profibrotic macrophages as monocyte-derived macrophages [39,40], that are characterized by a conserved transcriptional profile (SPP1, TREM2, MAFB, CD163, LGMN) [37,56] and are conventionally associated with M2-like polarization (mainly M2a and M2c) [47,61], although current data indicate that within the fibrotic niche they form a distinct scar-associated population that does not fit into the classical M1/M2 dichotomy. Following tissue injury, such macrophages do not fully acquire a mature tissue-resident phenotype but stably maintain a profibrotic state and secrete fibrogenic mediators such as TGF-β, RELMα, PDGF, and MMPs. Dynamic changes in macrophage subpopulation transcriptional programs determine the subsequent nature of tissue repair, driving a shift from normal regeneration to pathological remodeling.

### 3.2. Fibroblasts: Principal Effectors of Lung Remodeling

Stromal cells, particularly fibroblasts, are key effectors of both physiological and pathological tissue remodeling. In the lung, alveolar, adventitial, and peribronchial fibroblast subsets can be distinguished [64], with substantial phenotypic heterogeneity in both homeostasis and disease [65,66,67]. Fibroblast-to-myofibroblast differentiation is a central event in fibrosis irrespective of etiology [21]. Tissue fibroblasts and quiescent adventitial cells represent the major sources of myofibroblasts [24], although hematopoietic and perivascular cells, as well as epithelial and endothelial cells through epithelial–mesenchymal transition (EMT) and endothelial–mesenchymal transition (EndMT), respectively, may also acquire a myofibroblast phenotype [68,69]. In addition, monocyte/macrophage-to-mesenchymal transition (MMT) has been described, and myofibroblasts co-expressing macrophage markers have been identified in patients with pulmonary fibrosis [70,71,72,73].

Fibroblastic foci consist of centrally located myofibroblasts surrounded by activated fibroblasts. FAPα^+^ fibroblasts are among the earliest responders to tissue injury and, owing to their high proliferative capacity and ability to remodel the local microenvironment, can initiate formation of the pro-fibrotic niche and promote myofibroblast differentiation [74]. Lung injury gives rise to proliferative, stress-activated, inflammatory, and fibrotic fibroblast states [64]. Alveolar fibroblasts expressing *Pdgfra*, *Scube2*, and *Plin2* serve as a source of both inflammatory and fibrotic fibroblast populations in mice [64,75,76]. In humans, these cells sequentially transition toward an inflammatory phenotype (*ITGA8*+, *SFRP2*+, *CCL2*+ or *ITGA8*+, *SFRP4*+, *CXCL14*+) and subsequently, under the influence of TGF-β, toward a fibrotic *COL1A1*^high^ and *CTHRC1*+ state [64]. Markers of activated fibroblasts are broadly conserved between mice and humans; accordingly, *CTHRC1* and *COL1A1* are widely used to identify pro-fibrotic fibroblasts alongside the canonical myofibroblast marker α-smooth muscle actin (*α-SMA*) [21,65,67,77,78]. scRNA-seq and spatial transcriptomics have further revealed a population of *WNT5A*^+^*CTHRC1*^+^ myofibroblasts localized to both fibroblastic foci and areas of dense fibrosis [79]. TGF-β is a major inducer of fibroblast-to-myofibroblast differentiation [80], while the marked proliferative activity of these cells underlies the characterization of pulmonary fibrosis as a fibroproliferative disease [81]. Data summarizing contribution from various cell populations to the fibrotic lung remodeling are listed in Table 2.

## 4. IL-6 as a Central Mediator of Macrophage–Fibroblast Communication in Pulmonary Fibrosis

### 4.1. Modes of IL-6 Signaling

IL-6 is a pleiotropic cytokine that can exert either homeostatic or pathogenic effects depending on its concentration, duration of exposure, and signaling mode. Three distinct modes of IL-6 signaling have been described [83]. Classical signaling is initiated by binding of IL-6 to the membrane-bound IL-6 receptor α (IL-6Rα) together with the gp130 co-receptor on target cells [84]. In trans-signaling, soluble IL-6Rα (sIL-6Rα) is generated through ADAM10/17-mediated cleavage or alternative mRNA splicing. The IL-6-sIL-6Rα complex can subsequently activate virtually any cell expressing gp130 [85]. Under physiological conditions, IL-6 circulates at low concentrations together with sIL-6R, creating the potential for constitutive trans-signaling; however, this activity is restrained by a buffering system involving soluble gp130 (sgp130) [86]. A third mode, IL-6 trans-presentation, was demonstrated in a murine model of multiple sclerosis and involves interaction between gp130 on pathogenic Th17 cells and membrane-bound IL-6–IL-6Rα complexes displayed by dendritic cells [87]. More recently, macrophage-mediated trans-presentation of IL-6 to intestinal epithelial cells has also been described [88]. The biological effects of IL-6 are strongly influenced by the mode of signaling. Classical signaling is generally associated with anti-inflammatory, regenerative, and protective responses in target cells and tissues [89], whereas IL-6 trans-signaling and trans-presentation have been implicated in a range of pathological processes, including fibrosis [87,90,91,92]. In humans, IL-6 can be produced by both immune cells—including macrophages, neutrophils, dendritic cells, mast cells, B cells, and T cells—and non-immune cells, including fibroblasts, epithelial and endothelial cells, and myocytes [84,93]. IL-6Rα is likewise expressed by multiple immune and non-immune cell types, including two major effector populations in IPF, macrophages [94] and fibroblasts [95]. The gp130 co-receptor is expressed by most cell types, with particularly high expression in the spleen, lung, heart, and liver [96]. The expression pattern of IL-6 and its receptor, along with the modes of signaling, extend its effects beyond the cells that secrete it (immune and stromal cells) and target cells expressing the membrane-bound form of IL-6Rα. Crucially, it also affects cells that lack or express low levels of membrane-bound IL-6Rα yet express gp130. This latter mechanism is a prerequisite for the broad detrimental effects of IL-6.

### 4.2. IL-6 in Pathological Lung Remodeling

Fibrosis can arise as an outcome of chronic inflammation accompanied by sustained IL-6 production [97]. IL-6 levels are markedly elevated in IPF [98], CTD-ILD [18], and experimental mouse models of pulmonary fibrosis [99,100]. It was shown that IL-6 plays a pivotal role in different murine models of lung injury and fibrosis (Table 3).

Serum IL-6 concentrations in patients with interstitial lung diseases are almost an order of magnitude higher than those in healthy individuals [107], and higher IL-6 levels correlate with impaired pulmonary function and more severe disease [108]. IL-6 is also an important prognostic marker in IPF: each 1 pg/mL increase in serum IL-6 is associated with an increase of more than 5% in the risk of death [108]. A causal contribution of IL-6 to pulmonary fibrosis is further supported by attenuation of pathological changes following genetic IL-6-deficiency and by exacerbation of fibrosis through IL-6-dependent STAT3 activation [101,109,110].

The pro-fibrotic effects of IL-6 appear to be mediated predominantly through trans-signaling. Soluble IL-6Rα is elevated both in patients with IPF and in experimental models during early disease and subsequent progression [95,111]. In vivo neutralization of sIL-6Rα with recombinant gp130Fc attenuated inflammation and bleomycin-induced pulmonary fibrosis in mice and improved lung function [111]. Alveolar macrophages are likely to represent an important source of sIL-6Rα in fibrosis, as increased sIL-6Rα levels correlate with both alveolar macrophage abundance and ADAM17 activity in these cells [111].

IL-6 trans-signaling activates the IL-6/STAT3/Smad3 axis [112], thereby engaging TGF-β [112,113]. In response to TGF-β, alveolar fibroblasts acquire a fibrotic phenotype [64] through canonical SMAD2/3 signaling [114] or epigenetic regulation mediated by the transcription factor PU.1 [115]. Fibroblast-to-myofibroblast differentiation is a central event in pulmonary fibrotic remodeling because myofibroblasts are the principal effector cells responsible for scar formation [69,80]. The IL-6/STAT3/Smad3/TGF-β axis can also perpetuate inflammation through paracrine signaling from fibroblasts [95,112]. Activation of the TGF-β pathway by IL-6 may be mediated by Gremlin [95], which is overexpressed in IPF [116,117] and correlates with disease severity in mice [118]. Although some in vitro studies have shown an antagonistic effect of IL-6 on TGF-β [119], in the context of pulmonary fibrosis the interaction between IL-6 and TGF-β is synergistic.

Another mechanism implicated in fibrosis is epithelial fluidization mediated by the IL-6/SFK/YAP axis. Epithelial fluidization describes the transition of an epithelial layer from a mechanically stable to a more motile state, enabling collective cell migration and subsequent tissue reorganization. Fibroblast-derived IL-6 has been identified as a key driver of this process [102]. IL-6 also promotes fibrosis through direct effects on its major cellular effectors—macrophages and fibroblasts.

### 4.3. Pro-Fibrotic Effects of IL-6 on Macrophages

IL-6 can shift macrophage polarization toward a pro-fibrotic M2 phenotype. One mechanism through which IL-6 promotes M2 polarization involves regulation of iron metabolism [120]. Iron accumulation and altered iron handling are characteristic of IPF and are particularly prominent within fibrotic lesions [121]. IL-6 promotes intracellular iron accumulation in macrophages by inducing *HAMP* expression, which encodes hepcidin. Hepcidin binds the iron exporter ferroportin (FPN1), inducing its degradation and thereby suppressing intestinal iron absorption and macrophage iron recycling. The resulting impairment of systemic iron availability contributes to hypoferremia and iron retention within macrophages [122,123]. In pulmonary macrophages, IL-6 can also enhance iron sequestration through hepcidin-independent mechanisms, contributing to the progression of chronic obstructive pulmonary disease (COPD) and IPF [124]. IL-6-driven iron accumulation activates the HIF-1α/IL-10/STAT6 axis, promoting M2 polarization [125,126] while suppressing M1 responses [127]. Consistent with this mechanism, iron chelation reduces the abundance of M2 macrophages [128].

### 4.4. Pro-Fibrotic Effects of IL-6 on Fibroblasts

The pro-fibrotic activity of IL-6 is also mediated through direct effects on fibroblasts. IL-6 promotes fibroblast proliferation [95], induces fibroblast-to-myofibroblast differentiation [129], and enhances extracellular matrix protein production [111,130]. Notably, the increased proliferative activity of lung fibroblasts isolated from patients with IPF was inhibited by tocilizumab [95].

### 4.5. IL-6 and Age-Associated Changes in Fibrosis

The efficiency of tissue repair declines with age, making fibrosis an age-associated disease [21]. Cellular senescence is itself an important mechanism contributing to fibrotic remodeling in the lung [131,132,133]. For example, alveolar type 2 epithelial (AT2) cells with dysfunctional telomeres acquire a senescent phenotype and are found across several forms of fILD [18,82]. Epithelial cell senescence is considered a possible mechanism underlying impaired regeneration and acting as a trigger of fibrosis. In patients with IPF, senescence is observed not only in AT2 cells but also in pulmonary fibroblasts [134]. Xenotransplantation of senescent human lung fibroblasts into the lungs of immunodeficient mice was sufficient to induce progressive fibrosis accompanied by accumulation of senescent host cells [131]. In addition, soluble mediators released by M2 macrophages can induce senescence in stromal cells and thereby exacerbate fibrosis [62]. Thus, senescent cells within the lung parenchyma may actively contribute to the initiation and propagation of fibrotic remodeling.

Cellular senescence is characterized by stable cell-cycle arrest, epigenetic alterations, expansion of the lysosomal compartment, and acquisition of a senescence-associated secretory phenotype (SASP). The SASP includes increased production of IL-6, TGF-β, and MMPs [135,136,137]. The secretome of senescent human lung fibroblasts has been shown to exert profibrogenic effects both in vitro and in vivo [137,138,139]. Exposure of mouse embryonic fibroblasts (MEFs) to conditioned medium from senescent human lung fibroblasts induced their differentiation into myofibroblasts and increased expression of SASP components. Administration of the same conditioned medium into the lungs of immunodeficient mice increased hydroxyproline levels, a surrogate measure of collagen deposition and fibrosis severity [134]. Another hallmark of cellular aging is impaired mitochondrial homeostasis and function, which is also observed in alveolar macrophages in IPF [140]. Macrophages with dysfunctional mitochondria generate excessive ROS, thereby sustaining chronic inflammation [140] associated with fibrotic remodeling [141,142]. Chronic IL-6-driven inflammation contributes to multiple age-associated pathologies, leading to IL-6 being described as a gerokine [90]. Through the SASP and associated metabolic dysfunction, senescent cells sustain chronic inflammation and pro-fibrotic remodeling, positioning IL-6 as a central mechanistic link between cellular senescence, inflammaging, and fibrosis.

**Figure 2:**
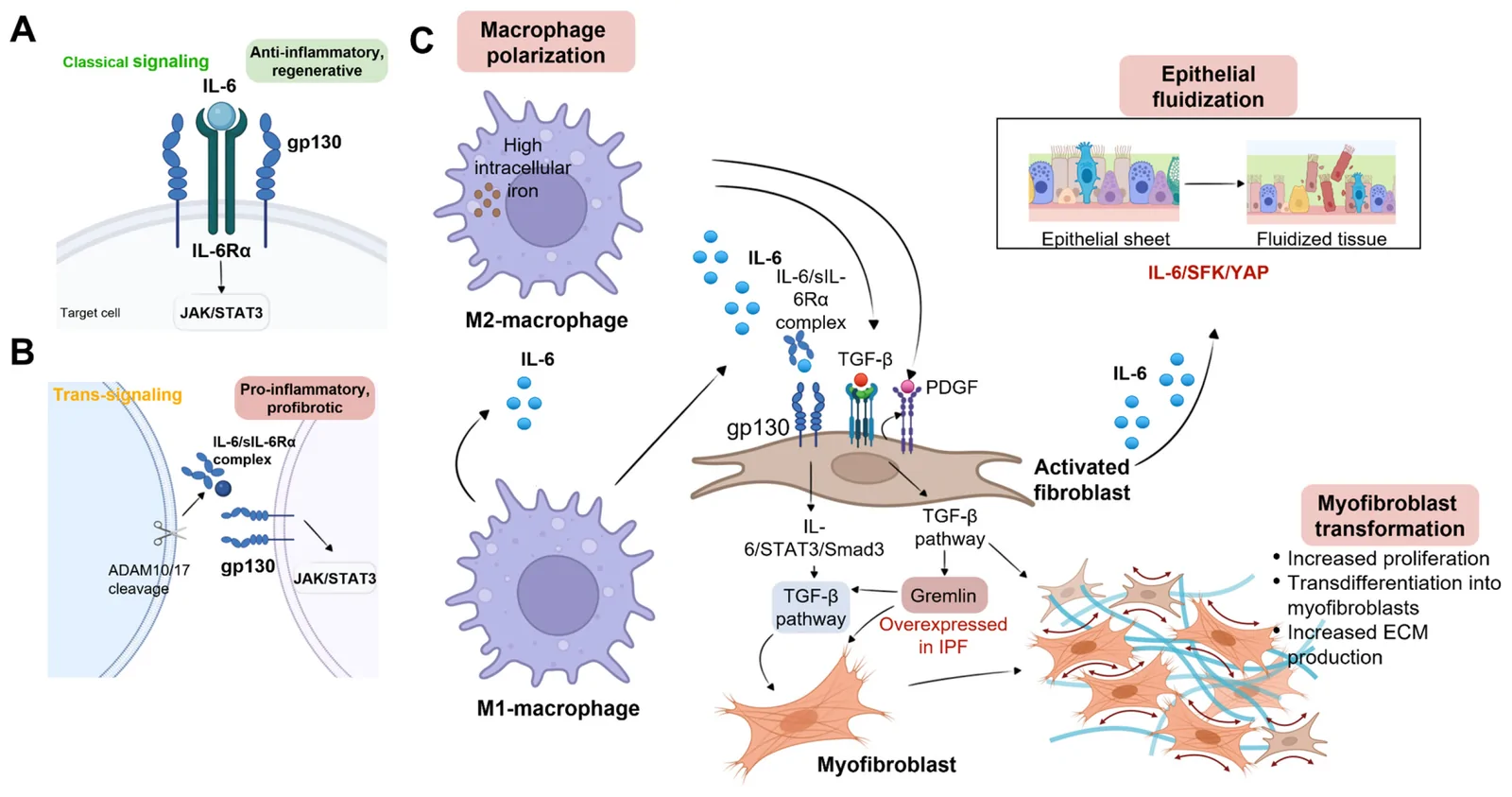
IL-6 as a mediator of macrophage–fibroblast interactions within the fibrotic niche. The effects of IL-6 depend on the mode of signaling, its concentration, and the duration of its production. Classical IL-6/IL-6Rα/gp130 signaling (**A**) is primarily associated with homeostatic functions, whereas trans-signaling through the IL-6/sIL-6Rα/gp130 complex (**B**) contributes to pathological tissue remodeling. The pro-fibrotic effects of IL-6 include M2 macrophage polarization, fibroblast activation and differentiation into myofibroblasts, and epithelial fluidization (**C**). IL-6-induced iron accumulation activates the HIF-1α/IL-10/STAT6 axis, thereby promoting M2 macrophage polarization. Through the IL-6/STAT3/Smad3 axis and Gremlin, IL-6 activates TGF-β-dependent fibroblast proliferation and fibroblast-to-myofibroblast transition. Epithelial fluidization is mediated by the IL-6/SFK/YAP axis and is characterized by a transition of the epithelium from a solid-like to a fluid-like state. Created in BioRender. Gubernatorova, E.O. (2026) https://BioRender.com/s3aeknh.

Overall, IL-6 emerges as a key mediator of pathological macrophage–fibroblast communication in fILD (Figure 2). Thus, monocyte-derived macrophages recruited by alveolar epithelial injury adopt an M1-like phenotype and produce a range of inflammatory mediators (including IL-6). They attract fibroblasts to the site of injury, regulating their migration, proliferation, and differentiation. In the early stages after injury, alveolar fibroblasts acquire an inflammatory phenotype in response to inflammatory cytokines [64]. Inflammatory fibroblasts probably represent an intermediate state preceding the appearance of profibrotic fibroblasts. The switch between fibroblast states is accompanied by changes in the expression of the corresponding markers [64,79]. The transition from the inflammatory to the fibrotic phenotype is induced by TGF-β. IL-6 acts as a mediator that, on the one hand, promotes the polarization of the macrophages themselves into a profibrotic phenotype [120], and on the other hand, supports the TGF-β signaling pathway in normal fibroblasts in a paracrine manner through the IL-6/STAT3/Smad3 axis [95]. IL-6 can be produced by both macrophages and fibroblasts. The broad spectrum of IL-6 effects appears to be achieved through IL-6 trans-signaling, which allows activation of not only fibroblasts but also in epithelial cells that express gp130 but lack membrane-bound IL-6 receptor. IL-6-dependent mechanisms establish a self-reinforcing circuit in which macrophages and fibroblasts mutually sustain and amplify fibrotic remodeling, making IL-6—particularly its trans-signaling pathway—not only a prognostic biomarker but also a compelling therapeutic target in fILD.

## 5. Clinical Trial Evidence for IL-6 Blockade in Fibrosis

Two drugs are currently approved for the treatment of IPF: nintedanib, a tyrosine kinase inhibitor targeting PDGFR, FGFR, and VEGFR, and pirfenidone, which inhibits TGF-β signaling [143,144]. Pirfenidone slows the decline in lung function, whereas nintedanib inhibits fibroblast proliferation; however, neither treatment halts disease progression or reduces mortality [145], underscoring the need for novel therapeutic strategies and providing a strong rationale for combination therapies. One promising approach is to disrupt pathogenic macrophage–fibroblast crosstalk, particularly its metabolic and senescence-associated components. Modulation of these processes has already shown therapeutic potential. For example, baicalein disrupted pathogenic cellular interactions and attenuated fibrosis by suppressing glycolysis and enhancing oxidative phosphorylation [146], whereas navitoclax alleviated pulmonary fibrosis induced by senescent human fibroblasts in immunodeficient mice [131].

Because IL-6 is a key mediator of macrophage–fibroblast communication in fibrosis, blockade of this pathway represents a potentially attractive therapeutic approach in fILD [147]. Clazakizumab and olokizumab directly neutralize IL-6, whereas tocilizumab [148], sarilumab, satralizumab, and levilimab target IL-6R. However, all of these agents act systemically, and given the highly pleiotropic nature of IL-6, selective inhibition of pathogenic trans-signaling with soluble gp130-based agents such as olamkicept may offer a more targeted strategy [149]. In vivo administration of recombinant gp130Fc reduced myofibroblast accumulation and decreased fibronectin and collagen deposition in the lungs of mice, thereby attenuating pulmonary fibrosis [111]. Direct clinical evidence supporting IL-6 blockade in pulmonary fibrosis remains limited. The efficacy of olokizumab is currently being evaluated in a phase 2/3, double-blind, parallel-group adaptive study in patients with progressive fILD (https://clinicaltrials.gov/study/NCT06440746, accessed on 1 August 2026), with study completion planned for 2028. In vitro, tocilizumab suppressed proliferation of lung fibroblasts derived from patients with IPF and inhibited the IL-6/STAT3/pSmad3 axis, thereby attenuating the fibrotic phenotype [95]. In a phase 3 randomized, double-blind, placebo-controlled trial, tocilizumab preserved lung function in patients with early systemic sclerosis-associated interstitial lung disease (SSc-ILD), a form of fILD [150]. Although tocilizumab slowed the progression of interstitial lung disease in systemic sclerosis, the overall certainty of the findings has been rated as low because much of the evidence was indirect and the number of studies was small, with limited sample sizes [151]. By contrast, its use during acute exacerbations of pulmonary fibrosis is not recommended because of the associated high mortality [152].

The limited efficacy of systemic IL-6 blockade in pulmonary fibrosis, despite substantial evidence implicating this cytokine in disease pathogenesis, may reflect the complex and incompletely understood etiology of the disease. Pulmonary fibrosis encompasses a spectrum of pathological processes [153], whereas currently available experimental models reproduce only selected aspects of human pathophysiology. Moreover, fibrosis in animal models is typically induced by acute injury and often resolves over time, in contrast to human IPF, which is chronic and relentlessly progressive [153]. In this review, we discussed the role of the immune system in the development of IPF and CTD-ILD, emphasizing that the active involvement of the adaptive immune response in the pathogenesis of CTD-ILD and the inflammatory nature of the disease provide the rationale for the efficacy of immunosuppressive therapy. This allows CTD-ILD to be regarded as a disease driven by chronic immune activation. IPF is considered an epithelium-driven disease [154], in which senescence contributes to impaired alveolar epithelial regeneration. The differences in the immune involvement requires different approaches to disease modeling. Many experimental studies use the classical or modified bleomycin model, which remains the standard model of pulmonary fibrosis. An alternative is LPS-induced pulmonary fibrosis [155,156,157]. Fundamental differences between bleomycin- and LPS-induced fibrosis in their mechanisms, kinetics, and translational relevance may in part reflect distinct contributions of the immune system, although combined LPS and bleomycin administration has been explored as an attempt to integrate the advantages of both models [158]. Nevertheless, comparative analysis of bleomycin- and LPS-induced fibrosis identified a shared *GPNMB*-expressing macrophage subset, suggesting the existence of a conserved macrophage response across these two models [159]. IL-6 is involved in all the fibrosis models mentioned; however, its effect may be bidirectional and stage-dependent. In particular, at the early stage of bleomycin injury, IL-6 may have a protective effect for the epithelium, and its blockade during this period may be harmful [103]. IL-6 has a protective acute effect but a pathological chronic effect [90,91,92]. Chronic pathology is orchestrated by trans-signaling and profibrotic changes [112]. The source of soluble forms of IL-6 receptor (sIL-6Rα) is presumed to be macrophages, as reflected in the correlation between increased ADAM17 activity in alveolar macrophages and sIL-6Rα content in fibrosis [111]. Thus, the shift from the reparative to the pathological effect of IL-6 is underlain by a context in which persistence of the signal extends beyond the reparative window and triggers trans-signaling, which broadens the range of target cells. This is consistent with the limited efficacy of systemic IL-6 blockade in the clinic and the need to take the time window into account. The narrow therapeutic window for IL-6 inhibition during the early inflammatory phase, together with functional redundancy among pro-inflammatory cytokines, may contribute to the inconsistent effects of targeting this pathway. Compensatory upregulation of other cytokines, including TNF, may require combined rather than isolated inhibition of IL-6 signaling [160,161,162] and may also provide alternative routes for fibroblast activation. Another important determinant of successful IL-6 blockade is targeting a specific cellular source. Immune cells are presumed to be the key source of IL-6 in both pathological remodeling and other lung diseases [163]. However, the stroma also produces IL-6. Which contribution is more critical for disease development remains to be elucidated. Nevertheless, it is already clear that the use of bispecific antibodies that enable blockade of cytokines of a specific cellular origin represents a promising direction for anticytokine therapy [164]. Such an approach takes into account the functional contribution of the cytokine from different cell populations, making it possible to target its pathological properties while minimizing off-target effects.

## 6. Conclusions

Pathological lung remodeling accompanied by fibrosis arises from a failure to maintain the balance between tissue repair and scarring, driven by chronic inflammation, cellular senescence, and metabolic reprogramming of key effector cells, particularly macrophages and fibroblasts. Communication between these populations is mediated by soluble factors, among which IL-6 occupies a central position. Despite compelling preclinical evidence and a clear association between elevated IL-6 levels and disease progression, clinical studies of systemic IL-6 blockade in pulmonary fibrosis have so far produced mixed results. These discrepancies may reflect the heterogeneity of interstitial lung diseases, limitations of currently available experimental models, a narrow therapeutic window for intervention, and compensatory activation of other pro-inflammatory cytokines. Future studies should therefore focus on refining our understanding of the pathogenesis of distinct fILD subtypes, developing and validating more clinically relevant experimental models, identifying biomarkers that can stratify patients most likely to benefit from anti-IL-6 therapy, and defining the spatiotemporal dynamics of IL-6-mediated interactions within the fibrotic niche. A more integrated understanding of IL-6-dependent intercellular communication may ultimately enable the development of more selective anti-fibrotic therapies capable of slowing the progression of pulmonary fibrosis.

## Figures and Tables

**Table 1:** Impact of different types of immune response on lung fibrosis pathogenesis. The major immune programs (Th1, Th2, Th17) and their associated cellular subsets, along with their pro-fibrotic and anti-fibrotic roles in pulmonary fibrosis, are summarized. The data illustrate the complex interplay between inflammatory and reparative immune responses that governs the progression or resolution of fibrotic disease. ILC2—Type 2 innate lymphoid cells. cDC2/DC3—Conventional dendritic cells type 2/type 3. GZMK—Granzyme K. NETs—Neutrophil extracellular traps. PAD4—Peptidyl arginine deiminase 4. DLCO—Diffusing capacity of the lungs for carbon monoxide. FVC—Forced vital capacity. NLR—Neutrophil-to-lymphocyte ratio. BAL—Bronchoalveolar lavage. AE-ILD—Acute exacerbation of interstitial lung disease. ASS-ILD—Antisynthetase syndrome-associated interstitial lung disease.

Type of Immune Response	Cells	Cytokines	Role in Fibrosis	Clinical Evidence	References
Th1 (intracellular pathogens)	Th1 cells	IL-1β, IL-6, IL-33, TNF, IL-12, IFN-y, MIG, IP10	Pro-fibrotic: In some contexts, Th1 cells may promote fibrosis through IFN-γ-driven chronic inflammation and fibroblast proliferation. Anti-fibrotic: IFN-γ-dependent CXCL9 production exerts anti-fibrotic effects in humans by suppressing fibroblast activation.	Higher baseline serum CXCL9 levels were associated with subsequent improvement in lung function and declined after treatment, suggesting that Th1-polarized responses may exert anti-fibrotic effects in a subset of IPF patients.	[35,36,37,38,39,40]
	CD8+ cytotoxic cells		Pro-fibrotic: In IPF, GZMK-high CD8+ T cells drive myofibroblast differentiation		
	NK cells		Pro-fibrotic: NK cells suppress collagen and αSMA in human. Anti-fibrotic: NK cells induce fibroblasts senescence in murine models.		
	M1-like macrophages		Pro-fibrotic: M1 macrophages drive early lung injury during fibrogenesis. Anti-fibrotic: M1 macrophages secrete matrix metalloproteinases and CXCL11, which restricts fibrosis through elimination of pro-fibrotic M2 macrophages.		
Th2 (extracellular pathogens, allergy)	Th2 cells	IL-4, IL-5, IL-6, IL-9, IL-13, IL-21, IL-24, IL-25	Pro-fibrotic: Th2 cells produce IL-4; may induce fibrosis after adoptive transfer.	Human ILC2s show increased IL1RL1/ST2 and decreased IFNGR1 in IPF. Human IPF tissue shows increased mast-cell density.	[41,42,43,44,45]
	ILC2		Pro-fibrotic: ILC2 induce collagen production, proliferation, and differentiation in fibroblasts.		
	M2-like macrophages		Pro-fibrotic: M2-like macrophages drive fibrosis through mesenchymal activation and contribute to the formation of a fibrotic niche		
	Mast cells		Pro-fibrotic: Mast cells cluster around fibroblast foci		
	cDC2/DC3		Pro-fibrotic: DC3-derived epiregulin drives fibroblast activation and sustains fibrosis. Anti-fibrotic: Blood DC subtypes are significantly reduced in IPF patients. The antifibrotic effect of dendritic cells is presumably due to their involvement in the inhibition of pro-inflammatory responses.		
Th17 (extracellular pathogens, fungi)	Th17	IL-17A, TGF-b, TNF, IL-6	Pro-fibrotic: Th17-derived IL-17A activates lung fibroblasts driving increased collagen I production.	Detectable IL-17A associated with lower lung function; the Th17/Treg ratio inversely correlated with DLCO in ASS-ILD. Raised blood neutrophils/NLR associate with FVC decline; BAL neutrophilia independently predicts poor 90-day survival in AE-ILD	[46,47,48,49,50,51,52,53,54]
	Basophils		Pro-fibrotic: Basophils may recruit Th17 cells		
	γδ T17 cells		Pro-fibrotic: γδ T17 cells are key source of IL-17.		
	Neutrophils		Pro-fibrotic: NET-like structures occur in IPF lung/BAL; PAD4 deficiency reduces experimental fibrosis.		
	Inflammatory monocytes		Pro-fibrotic: Monocyte-derived alveolar macrophages localise near fibroblasts.		

**Table 2:** Contribution of different cell types to fibrotic niche formation. The key cellular constituents of the fibrotic microenvironment in the lung include injured epithelial cells, macrophage subsets (interstitial, M1-like, M2-like), fibroblast populations (activated myofibroblasts and inflammatory fibroblasts), recruited monocytes, and endothelial/perivascular cells. The data highlight the complex cellular crosstalk driven by cytokines, growth factors, and extracellular matrix remodeling that sustains persistent fibrosis and tissue destruction.

Cellular Component	Features	Major Pro-Fibrotic Functions	Interactions Within the Niche	References
Injured alveolar epithelial cells	Necrotic death and cellular senescence of alveolar epithelial cells, which serve as sources of DAMPs (IL-33, HMGB1, ATP)	DAMPs induce a pro-inflammatory M1 response. Senescent epithelial cells exhibit impaired repair and, through the SASP, lead to the accumulation of senescent cells in the pulmonary microenvironment, thereby creating a pro-fibrotic milieu	Recruit and activate macrophages; contribute to pro-inflammatory state	[16,17,18,82]
Interstitial macrophages	CD11b^+^, CD11c^+^, CD169^+^ cells; anatomically localized within alveolar septa and peribronchial/perivascular interstitium	Regulate local inflammation, antigen presentation and stromal responses; may acquire pro-fibrotic functions depending on microenvironmental signals (e.g., TGF-β, IL-13)	Activate interstitial fibroblasts; amplify local TGF-β-driven responses; may serve as precursors to macrophages associated with idiopathic pulmonary fibrosis	[22,35,36]
Recruited monocytes	*SPP1*+, *MAFB*+, *CD163*+, and *LGMN*+	LGMN^+^ macrophages degrade type IV collagen, creating space for type I collagen deposition, directly promoting fibrosis. Produce TGF-β1, CCL2, SPP1, and other cytokines that sustain fibroblast activation and recruit additional inflammatory cells	Interact with fibroblasts and myofibroblasts via TGF-β and SPP1 signaling, reinforcing a positive feedback loop that perpetuates fibrosis	[21,24]
M1-like inflammatory macrophages	iNOS^+^, MHC-II^high^, CD80^+^, CD86^+^	Secrete IL-1β, TNF-α, IL-6, ROS; promote early injury and inflammatory-cell recruitment; their effects are context dependent and can include anti-fibrotic activity	Amplify epithelial injury and initiate inflammatory signalling; may transit to M2-like	[38,47,50,73]
M2-like pro-fibrotic macrophages	CD163^+^, CD206^+^, Arg1^+^, CD36^+^, ^+^, CD9^+^	Promote tissue remodelling through production of TGF-β, IL-10, other growth factors, ROS and other pro-fibrotic mediators	Reinforce fibroblast activation and suppress anti-fibrotic stromal transcription programs	[47,58,61,62]
Activated fibroblasts and myofibroblasts	*COL1A1*, *CTHRC1*, *α-SMA*; matrix-producing phenotype	Produce collagen, fibronectin and other ECM components	Respond to macrophage-derived IL-6, TGF-β and PDGF	[21,65,67,78,80]
Inflammatory fibroblasts	*ITGA8*+, *SFRP2*+, *CCL2*+ or *ITGA8*+, *SFRP4*+, *CXCL14*+	Facilitate early formation of the pro-fibrotic niche	Promote myofibroblast differentiation	[67,80,81]
Endothelial and perivascular cells	Endothelial-to-mesenchymal transition (EndMT): loss of endothelial markers (CD31, VE-cadherin), gain of mesenchymal/myofibroblast markers (α-SMA, FSP-1, fibronectin, collagen I)	May contribute to stromal expansion, altered vascular microenvironment and persistent injury	Interact with macrophages and fibroblasts in perivascular regions; may generate mesenchymal-like cells	[68,69]

**Table 3:** Role of IL-6 in murine models of lung injury and fibrosis. The effects of genetic depletion (IL-6^KO/KO^) or pharmacological inactivation (neutralizing anti-IL-6 or anti-IL-6R antibodies) of IL-6 signaling in various mouse models of lung injury and fibrosis. The data reveal a context-dependent and biphasic role for IL-6, with early blockade exacerbating fibrosis in some models and late blockade attenuating disease progression, highlighting the importance of temporal dynamics in targeting IL-6 for anti-fibrotic therapy. BLM—bleomycin; IL-6^KO/KO^ mice—homozygous IL-6 knockout mice (completely deficient in IL-6 production); ADA^KO/KO^ mice—homozygous adenosine deaminase (ADA)-deficient mice.

Mouse Model	IL-6 Inhibition/Depletion	Experimental Effects	References
BLM (1 mg/kg)	IL-6 ^KO/KO^ mice	Two days after BLM treatment: Attenuated number of cells, including macrophages and neutrophils in BAL fluid, decreased TGF-β and CCL3 and lung inflammatory cell accumulation in IL-6 ^KO/KO^ mice. Three weeks after BLM treatment: Attenuated fibrotic changes and decreased collagen deposition, no difference in TGF-β, lower levels of CCL3 in IL-6 ^KO/KO^ mice.	[101,102]
BLM (3 mg/kg)	Anti-IL-6 treatment	Pharmacological IL-6 blockade at 0.5 to 3 days post-instillation (dpi) enhanced apoptosis of type 2 pneumocytes and neutrophilic infiltration and markedly accelerated fibrosis in the lung. In contrast, inhibition at 8 to 10 dpi by IL-6-neutralizing antibody ameliorated lung fibrosis.	[103]
ADA-deficiency model of chronic lung disease	IL-6^KO/KO^ ADA^KO/KO^ mice	Both pharmacologic blockade and genetic ablation of IL-6 attenuated pulmonary inflammation, remodeling and fibrosis in this model.	[104]
C-iron irradiation of lung	IL-6^KO/KO^	Attenuated fibrotic changes and tissue remodeling in IL-6 ^KO/KO^ mice. IL-6 plays an important role for fibrotic changes in the alveolar wall after irradiation.	[105]
Radiation pneumonia	Early or prolonged IL-6R blockade	No effect on lung damage	[106]

## Data Availability

No new data were created or analyzed in this study. Data sharing is not applicable to this article.

## References

[1] Lurje I., Gaisa N.T., Weiskirchen R., Tacke F. (2023). Mechanisms of Organ Fibrosis: Emerging Concepts and Implications for Novel Treatment Strategies. Mol. Asp. Med..

[2] Murtha L.A., Schuliga M.J., Mabotuwana N.S., Hardy S.A., Waters D.W., Burgess J.K., Knight D.A., Boyle A.J. (2017). The Processes and Mechanisms of Cardiac and Pulmonary Fibrosis. Front. Physiol..

[3] Yu H.-X., Feng Z., Lin W., Yang K., Liu R.-Q., Li J.-Q., Liu X.-Y., Pei M., Yang H.-T. (2022). Ongoing Clinical Trials in Aging-Related Tissue Fibrosis and New Findings Related to AhR Pathways. Aging Dis..

[4] Enomoto N. (2024). Relationship between Idiopathic Interstitial Pneumonias (IIPs) and Connective Tissue Disease-Related Interstitial Lung Disease (CTD-ILD): A Narrative Review. Respir. Investig..

[5] Kamiya M., Carter H., Espindola M.S., Doyle T.J., Lee J.S., Merriam L.T., Zhang F., Kawano-Dourado L., Sparks J.A., Hogaboam C.M. (2024). Immune Mechanisms in Fibrotic Interstitial Lung Disease. Cell.

[6] Lederer D.J., Martinez F.J. (2018). Idiopathic Pulmonary Fibrosis. N. Engl. J. Med..

[7] Wang J., Zhu F., Liu Y., Luo R., Fan Z., Dai W., Wei S., Lin C. (2025). A Novel Mouse Model of Pulmonary Fibrosis: Twice-Repeated Oropharyngeal Bleomycin Administration Mimicking Human Pathology. Biochem. Cell Biol..

[8] Ezzo M., Spindler K., Wang J.B., Lee D., Pecoraro G., Cowen J., Pakshir P., Hinz B. (2024). Acute Contact with Profibrotic Macrophages Mechanically Activates Fibroblasts via αvβ3 Integrin-Mediated Engagement of Piezo1. Sci. Adv..

[9] Lodyga M., Cambridge E., Karvonen H.M., Pakshir P., Wu B., Boo S., Kiebalo M., Kaarteenaho R., Glogauer M., Kapoor M. (2019). Cadherin-11-Mediated Adhesion of Macrophages to Myofibroblasts Establishes a Profibrotic Niche of Active TGF-β. Sci. Signal..

[10] Li Y., Zhao J., Yin Y., Li K., Zhang C., Zheng Y. (2022). The Role of IL-6 in Fibrotic Diseases: Molecular and Cellular Mechanisms. Int. J. Biol. Sci..

[11] Astrab L.R., Skelton M.L., Caliari S.R. (2025). M2 Macrophage Co-Culture Overrides Viscoelastic Hydrogel Mechanics to Promote IL6-Dependent Fibroblast Activation. Cell Biomater..

[12] Budinger G.R.S. (2025). Alveolar Macrophage-Fibroblast Crosstalk Includes a Feedforward Arginase 1/IL-6 Circuit Implicated in Pulmonary Fibrosis. J. Clin. Investig..

[13] McCowan J., Fercoq F., Kirkwood P.M., T’Jonck W., Hegarty L.M., Mawer C.M., Cunningham R., Mirchandani A.S., Hoy A., Humphries D.C. (2021). The Transcription Factor EGR2 Is Indispensable for Tissue-Specific Imprinting of Alveolar Macrophages in Health and Tissue Repair. Sci. Immunol..

[14] Woo Y.D., Jeong D., Chung D.H. (2021). Development and Functions of Alveolar Macrophages. Mol. Cells.

[15] Joshi N., Watanabe S., Verma R., Jablonski R.P., Chen C.-I., Cheresh P., Markov N.S., Reyfman P.A., McQuattie-Pimentel A.C., Sichizya L. (2020). A Spatially Restricted Fibrotic Niche in Pulmonary Fibrosis Is Sustained by M-CSF/M-CSFR Signalling in Monocyte-Derived Alveolar Macrophages. Eur. Respir. J..

[16] Burgoyne R.A., Fisher A.J., Borthwick L.A. (2021). The Role of Epithelial Damage in the Pulmonary Immune Response. Cells.

[17] Ishikawa G., Liu A., Herzog E.L. (2021). Evolving Perspectives on Innate Immune Mechanisms of IPF. Front. Mol. Biosci..

[18] Lee J.H., Jang J.H., Lee S., Her M. (2025). Immunopathogenic Mechanisms in Connective Tissue Disease-Associated Interstitial Lung Disease: Incessant Loop of Immunity to Fibrosis. Int. J. Mol. Sci..

[19] Hata K., Yanagihara T., Matsubara K., Kunimura K., Suzuki K., Tsubouchi K., Eto D., Ando H., Uehara M., Ikegame S. (2023). Mass Cytometry Identifies Characteristic Immune Cell Subsets in Bronchoalveolar Lavage Fluid from Interstitial Lung Diseases. Front. Immunol..

[20] Raghu G., Anstrom K.J., King T.E., Lasky J.A., Martinez F.J. (2012). Prednisone, Azathioprine, and N-Acetylcysteine for Pulmonary Fibrosis. N. Engl. J. Med..

[21] Bhattacharya M., Ramachandran P. (2023). Immunology of Human Fibrosis. Nat. Immunol..

[22] Behmoaras J., Mulder K., Ginhoux F., Petretto E. (2025). The Spatial and Temporal Activation of Macrophages during Fibrosis. Nat. Rev. Immunol..

[23] Adamo L., Rocha-Resende C., Prabhu S.D., Mann D.L. (2020). Reappraising the Role of Inflammation in Heart Failure. Nat. Rev. Cardiol..

[24] Di X., Chen J., Li Y., Wang M., Wei J., Li T., Liao B., Luo D. (2024). Crosstalk between Fibroblasts and Immunocytes in Fibrosis: From Molecular Mechanisms to Clinical Trials. Clin. Transl. Med..

[25] Vu T.N., Chen X., Foda H.D., Smaldone G.C., Hasaneen N.A. (2019). Interferon-γ Enhances the Antifibrotic Effects of Pirfenidone by Attenuating IPF Lung Fibroblast Activation and Differentiation. Respir. Res..

[26] Kim J.H., Kim H.Y., Kim S., Chung J.-H., Park W.S., Chung D.H. (2005). Natural Killer T (NKT) Cells Attenuate Bleomycin-Induced Pulmonary Fibrosis by Producing Interferon-Gamma. Am. J. Pathol..

[27] Hasaneen N., Haley J., Foda H., Condos R., Smaldone G. (2016). Anti-Fibrotic Role of Inhaled Interferon-γ Detected by Proteomic and Genomic Studies in Patients with Idiopathic Pulmonary Fibrosis. QJM Int. J. Med..

[28] Mack M. (2018). Inflammation and Fibrosis. Matrix Biol..

[29] Di X., Gao X., Peng L., Ai J., Jin X., Qi S., Li H., Wang K., Luo D. (2023). Cellular Mechanotransduction in Health and Diseases: From Molecular Mechanism to Therapeutic Targets. Signal Transduct. Target. Ther..

[30] Yasui M., Miyazaki Y., Mitaka K., Ishizuka M., Unoura K., Tamaoka M., Sumi Y., Inase N. (2012). Epithelial-Mesenchymal Transition in Chronic Hypersensitivity Pneumonitis. J. Med. Dent. Sci..

[31] Quan T.E., Cowper S.E., Bucala R. (2006). The Role of Circulating Fibrocytes in Fibrosis. Curr. Rheumatol. Rep..

[32] Willis B.C., duBois R.M., Borok Z. (2006). Epithelial Origin of Myofibroblasts during Fibrosis in the Lung. Proc. Am. Thorac. Soc..

[33] Senoo S., Higo H., Taniguchi A., Kiura K., Maeda Y., Miyahara N. (2023). Pulmonary Fibrosis and Type-17 Immunity. Respir. Investig..

[34] Wilson M.S., Madala S.K., Ramalingam T.R., Gochuico B.R., Rosas I.O., Cheever A.W., Wynn T.A. (2010). Bleomycin and IL-1beta-Mediated Pulmonary Fibrosis Is IL-17A Dependent. J. Exp. Med..

[35] Kuang H., Chen Y., Yang X., Sun R., Chen Y., Hu Z., Ye L., Deng C., Li X., Wu J. (2026). Macrophage Polarization as a Therapeutic Target in Lung Diseases: From Pathogenesis to Precision Intervention. Int. Immunopharmacol..

[36] Tan S.Y.S., Krasnow M.A. (2016). Developmental Origin of Lung Macrophage Diversity. Development.

[37] Cui H., Banerjee S., Xie N., Hussain M., Jaiswal A., Liu H., Kulkarni T., Antony V.B., Liu R.-M., Colonna M. (2025). TREM2 Promotes Lung Fibrosis via Controlling Alveolar Macrophage Survival and Pro-Fibrotic Activity. Nat. Commun..

[38] Ge Z., Chen Y., Ma L., Hu F., Xie L. (2024). Macrophage Polarization and Its Impact on Idiopathic Pulmonary Fibrosis. Front. Immunol..

[39] Misharin A.V., Morales-Nebreda L., Reyfman P.A., Cuda C.M., Walter J.M., McQuattie-Pimentel A.C., Chen C.-I., Anekalla K.R., Joshi N., Williams K.J.N. (2017). Monocyte-Derived Alveolar Macrophages Drive Lung Fibrosis and Persist in the Lung over the Life Span. J. Exp. Med..

[40] Satoh T., Nakagawa K., Sugihara F., Kuwahara R., Ashihara M., Yamane F., Minowa Y., Fukushima K., Ebina I., Yoshioka Y. (2017). Identification of an Atypical Monocyte and Committed Progenitor Involved in Fibrosis. Nature.

[41] Acharya P.S., Majumdar S., Jacob M., Hayden J., Mrass P., Weninger W., Assoian R.K., Puré E. (2008). Fibroblast Migration Is Mediated by CD44-Dependent TGF Beta Activation. J. Cell Sci..

[42] Clark R.A., McCoy G.A., Folkvord J.M., McPherson J.M. (1997). TGF-Beta 1 Stimulates Cultured Human Fibroblasts to Proliferate and Produce Tissue-like Fibroplasia: A Fibronectin Matrix-Dependent Event. J. Cell. Physiol..

[43] Fine A., Goldstein R.H. (1987). The Effect of Transforming Growth Factor-Beta on Cell Proliferation and Collagen Formation by Lung Fibroblasts. J. Biol. Chem..

[44] Knipper J.A., Willenborg S., Brinckmann J., Bloch W., Maaß T., Wagener R., Krieg T., Sutherland T., Munitz A., Rothenberg M.E. (2015). Interleukin-4 Receptor α Signaling in Myeloid Cells Controls Collagen Fibril Assembly in Skin Repair. Immunity.

[45] Alvarez R.H., Kantarjian H.M., Cortes J.E. (2006). Biology of Platelet-Derived Growth Factor and Its Involvement in Disease. Mayo Clin. Proc..

[46] Krenkel O., Puengel T., Govaere O., Abdallah A.T., Mossanen J.C., Kohlhepp M., Liepelt A., Lefebvre E., Luedde T., Hellerbrand C. (2018). Therapeutic Inhibition of Inflammatory Monocyte Recruitment Reduces Steatohepatitis and Liver Fibrosis. Hepatology.

[47] Gharavi A.T., Hanjani N.A., Movahed E., Doroudian M. (2022). The Role of Macrophage Subtypes and Exosomes in Immunomodulation. Cell. Mol. Biol. Lett..

[48] Kishore A., Petrek M. (2021). Roles of Macrophage Polarization and Macrophage-Derived miRNAs in Pulmonary Fibrosis. Front. Immunol..

[49] Mahalanobish S., Saha S., Dutta S., Sil P.C. (2020). Matrix Metalloproteinase: An Upcoming Therapeutic Approach for Idiopathic Pulmonary Fibrosis. Pharmacol. Res..

[50] Zheng Y., Fan G., Xiao Y., Peng Y., Hu M., Shen S., Liu S., Meng Z., Zhang R. (2025). The Role of ELK4 Up-Regulation in Macrophage Polarization and Its Mechanism in Connective Tissue Disease-Associated Interstitial Lung Disease. Int. Immunopharmacol..

[51] Zhang Y., Lin J., Han X., Chen X. (2025). Saroglitazar Ameliorates Pulmonary Fibrosis Progression in Mice by Suppressing NF-κB Activation and Attenuating Macrophage M1 Polarization. Medicina.

[52] Rennard S.I., Hunninghake G.W., Bitterman P.B., Crystal R.G. (1981). Production of Fibronectin by the Human Alveolar Macrophage: Mechanism for the Recruitment of Fibroblasts to Sites of Tissue Injury in Interstitial Lung Diseases. Proc. Natl. Acad. Sci. USA.

[53] Tsukamoto Y., Helsel W.E., Wahl S.M. (1981). Macrophage Production of Fibronectin, a Chemoattractant for Fibroblasts. J. Immunol..

[54] Damm E.W., Clements W.K. (2017). PDGF Signaling Guides Neural Crest Contribution to the Haematopoietic Stem Cell Specification Niche. Nat. Cell Biol..

[55] Nosenko M.A., Moysenovich A.M., Arkhipova A.Y., Atretkhany K.N., Nedospasov S.A., Drutskaya M.S., Moisenovich M.M. (2021). Fibroblasts Upregulate Expression of Adhesion Molecules and Promote Lymphocyte Retention in 3D Fibroin/Gelatin Scaffolds. Bioact. Mater..

[56] Morse C., Tabib T., Sembrat J., Buschur K.L., Bittar H.T., Valenzi E., Jiang Y., Kass D.J., Gibson K., Chen W. (2019). Proliferating SPP1/MERTK-expressing macrophages in idiopathic pulmonary fibrosis. Eur. Respir. J..

[57] Wendisch D., Dietrich O., Mari T., von Stillfried S., Ibarra I.L., Mittermaier M., Mache C., Chua R.L., Knoll R., Timm S. (2021). SARS-CoV-2 Infection Triggers Profibrotic Macrophage Responses and Lung Fibrosis. Cell.

[58] Huang W.-C., Sala-Newby G.B., Susana A., Johnson J.L., Newby A.C. (2012). Classical Macrophage Activation Up-Regulates Several Matrix Metalloproteinases through Mitogen Activated Protein Kinases and Nuclear Factor-κB. PLoS ONE.

[59] Kim J.-Y., Cho D.-W., Choi J.-Y., Jeong S., Kang M., Kim W.J., Hong I.-S., Song H., Chang H., Yang S.-R. (2024). CXCL11 Reprograms M2-Biased Macrophage Polarization to Alleviate Pulmonary Fibrosis in Mice. Cell Biosci..

[60] Zhang R., Jiang Q., Gao S., Zhang H., Xia Q., Liu B., Zhu J., Jiang H., Zhao R., Dong H. (2024). Favipiravir Ameliorates Bleomycin-Induced Pulmonary Fibrosis by Reprogramming M1/M2 Macrophage Polarization. Int. Immunopharmacol..

[61] Ji W.-J., Ma Y.-Q., Zhou X., Zhang Y.-D., Lu R.-Y., Sun H.-Y., Guo Z.-Z., Zhang Z., Li Y.-M., Wei L.-Q. (2014). Temporal and Spatial Characterization of Mononuclear Phagocytes in Circulating, Lung Alveolar and Interstitial Compartments in a Mouse Model of Bleomycin-Induced Pulmonary Injury. J. Immunol. Methods.

[62] Dyachkova U., Vigovskiy M., Basalova N., Efimenko A., Grigorieva O. (2023). M2-Macrophage-Induced Chronic Inflammation Promotes Reversible Mesenchymal Stromal Cell Senescence and Reduces Their Anti-Fibrotic Properties. Int. J. Mol. Sci..

[63] Nataliya B., Mikhail A., Vladimir P., Olga G., Maksim V., Ivan Z., Ekaterina N., Georgy S., Natalia D., Pavel M. (2023). Mesenchymal Stromal Cells Facilitate Resolution of Pulmonary Fibrosis by miR-29c and miR-129 Intercellular Transfer. Exp. Mol. Med..

[64] Tsukui T., Wolters P.J., Sheppard D. (2024). Alveolar Fibroblast Lineage Orchestrates Lung Inflammation and Fibrosis. Nature.

[65] Buechler M.B., Pradhan R.N., Krishnamurty A.T., Cox C., Calviello A.K., Wang A.W., Yang Y.A., Tam L., Caothien R., Roose-Girma M. (2021). Cross-Tissue Organization of the Fibroblast Lineage. Nature.

[66] Narvaez Del Pilar O., Gacha Garay M.J., Chen J. (2022). Three-Axis Classification of Mouse Lung Mesenchymal Cells Reveals Two Populations of Myofibroblasts. Development.

[67] Tsukui T., Sun K.-H., Wetter J.B., Wilson-Kanamori J.R., Hazelwood L.A., Henderson N.C., Adams T.S., Schupp J.C., Poli S.D., Rosas I.O. (2020). Collagen-Producing Lung Cell Atlas Identifies Multiple Subsets with Distinct Localization and Relevance to Fibrosis. Nat. Commun..

[68] Friedman S.L., Sheppard D., Duffield J.S., Violette S. (2013). Therapy for Fibrotic Diseases: Nearing the Starting Line. Sci. Transl. Med..

[69] Pakshir P., Noskovicova N., Lodyga M., Son D.O., Schuster R., Goodwin A., Karvonen H., Hinz B. (2020). The Myofibroblast at a Glance. J. Cell Sci..

[70] Li X., Liu Y., Tang Y., Xia Z. (2024). Transformation of Macrophages into Myofibroblasts in Fibrosis-Related Diseases: Emerging Biological Concepts and Potential Mechanism. Front. Immunol..

[71] Vierhout M., Ayoub A., Naiel S., Yazdanshenas P., Revill S.D., Reihani A., Dvorkin-Gheva A., Shi W., Ask K. (2021). Monocyte and Macrophage Derived Myofibroblasts: Is It Fate? A Review of the Current Evidence. Wound Repair Regen..

[72] Wei Y., Guo H., Yang X., Tang X.X. (2026). Macrophage-to-Myofibroblast Transdifferentiation Contributes to Pulmonary Fibrosis via the MERTK-SPP1-SRC-TKS5 Signaling Axis. Adv. Sci. Weinh. Baden-Wurtt. Ger..

[73] Zhang J., Huang J., Yang Q., Zeng L., Deng K. (2024). Regulatory Mechanisms of Macrophage-Myofibroblast Transdifferentiation: A Potential Therapeutic Strategy for Fibrosis. Biochem. Biophys. Res. Commun..

[74] Basalova N.A., Vigovskiy M.A., Popov V.S., Lagereva E.A., Grigorieva O.A., Efimenko A.Y. (2024). The Role of Activated Stromal Cells in Fibrotic Foci Formation and Reversion. Cells.

[75] Barkauskas C.E., Cronce M.J., Rackley C.R., Bowie E.J., Keene D.R., Stripp B.R., Randell S.H., Noble P.W., Hogan B.L.M. (2013). Type 2 Alveolar Cells Are Stem Cells in Adult Lung. J. Clin. Investig..

[76] McGowan S.E., Torday J.S. (1997). The Pulmonary Lipofibroblast (Lipid Interstitial Cell) and Its Contributions to Alveolar Development. Annu. Rev. Physiol..

[77] Melms J.C., Biermann J., Huang H., Wang Y., Nair A., Tagore S., Katsyv I., Rendeiro A.F., Amin A.D., Schapiro D. (2021). A Molecular Single-Cell Lung Atlas of Lethal COVID-19. Nature.

[78] Ruiz-Villalba A., Romero J.P., Hernández S.C., Vilas-Zornoza A., Fortelny N., Castro-Labrador L., San Martin-Uriz P., Lorenzo-Vivas E., García-Olloqui P., Palacio M. (2020). Single-Cell RNA Sequencing Analysis Reveals a Crucial Role for CTHRC1 (Collagen Triple Helix Repeat Containing 1) Cardiac Fibroblasts After Myocardial Infarction. Circulation.

[79] Watanabe N., Yoshida M., Hirano Y., Fujimoto S., Matsubayashi S., Ishiguro T., Takahashi N., Shimizu Y., Takayanagi N., Kawabata Y. (2025). Integrated Spatial and Single-Cell Transcriptomics Reveal PAK Kinase as a Therapeutic Target in Fibroblastic Foci and Dense Fibrosis of Idiopathic Pulmonary Fibrosis. Eur. Respir. J..

[80] Ju X., Wang K., Wang C., Zeng C., Wang Y., Yu J. (2024). Regulation of Myofibroblast Dedifferentiation in Pulmonary Fibrosis. Respir. Res..

[81] Molina C., Tsukui T., Khan I.S., Ren X., Qiu W., Matthay M., Wolters P., Sheppard D. (2025). Clonal Expansion of Alveolar Fibroblast Progeny Drives Pulmonary Fibrosis in Mouse Models. J. Clin. Investig..

[82] Parimon T., Chen P., Stripp B.R., Liang J., Jiang D., Noble P.W., Parks W.C., Yao C. (2023). Senescence of Alveolar Epithelial Progenitor Cells: A Critical Driver of Lung Fibrosis. Am. J. Physiol. Cell Physiol..

[83] Drutskaya M.S., Gogoleva V.S., Atretkhany K.-S.N., Gubernatorova E.O., Zvartsev R.V., Nosenko M.A., Nedospasov S.A. (2018). [Proinflammatory and Immunoregulatory Functions of Interleukin 6 as Identified by Reverse Genetics]. Mol. Biol..

[84] Hunter C.A., Jones S.A. (2015). IL-6 as a Keystone Cytokine in Health and Disease. Nat. Immunol..

[85] Lust J.A., Donovan K.A., Kline M.P., Greipp P.R., Kyle R.A., Maihle N.J. (1992). Isolation of an mRNA Encoding a Soluble Form of the Human Interleukin-6 Receptor. Cytokine.

[86] Baran P., Hansen S., Waetzig G.H., Akbarzadeh M., Lamertz L., Huber H.J., Ahmadian M.R., Moll J.M., Scheller J. (2018). The Balance of Interleukin (IL)-6, IL-6·soluble IL-6 Receptor (sIL-6R), and IL-6·sIL-6R·sgp130 Complexes Allows Simultaneous Classic and Trans-Signaling. J. Biol. Chem..

[87] Heink S., Yogev N., Garbers C., Herwerth M., Aly L., Gasperi C., Husterer V., Croxford A.L., Möller-Hackbarth K., Bartsch H.S. (2017). Trans-Presentation of IL-6 by Dendritic Cells Is Required for the Priming of Pathogenic TH17 Cells. Nat. Immunol..

[88] Gong F., Zhang Q., Qian J., He Y., Du K., Fang H., Li Q., Yang Y., Wei H.-X., Jin T. (2026). Trans-Presentation of IL-6Rα by Macrophages Drives Colorectal Cancer Development. Cell Rep..

[89] Fuster J.J., Walsh K. (2014). The Good, the Bad, and the Ugly of Interleukin-6 Signaling. EMBO J..

[90] Forcina L., Franceschi C., Musarò A. (2022). The Hormetic and Hermetic Role of IL-6. Ageing Res. Rev..

[91] Galván-Peña S., O’Neill L.A.J. (2014). Metabolic Reprograming in Macrophage Polarization. Front. Immunol..

[92] Tanaka T., Narazaki M., Masuda K., Kishimoto T. (2016). Regulation of IL-6 in Immunity and Diseases. Adv. Exp. Med. Biol..

[93] Rincon M., Irvin C.G. (2012). Role of IL-6 in Asthma and Other Inflammatory Pulmonary Diseases. Int. J. Biol. Sci..

[94] Wognum A.W., van Gils F.C., Wagemaker G. (1993). Flow Cytometric Detection of Receptors for Interleukin-6 on Bone Marrow and Peripheral Blood Cells of Humans and Rhesus Monkeys. Blood.

[95] Epstein Shochet G., Brook E., Bardenstein-Wald B., Shitrit D. (2020). TGF-β Pathway Activation by Idiopathic Pulmonary Fibrosis (IPF) Fibroblast Derived Soluble Factors Is Mediated by IL-6 Trans-Signaling. Respir. Res..

[96] Silver J.S., Hunter C.A. (2010). Gp130 at the Nexus of Inflammation, Autoimmunity, and Cancer. J. Leukoc. Biol..

[97] Fielding C.A., Jones G.W., McLoughlin R.M., McLeod L., Hammond V.J., Uceda J., Williams A.S., Lambie M., Foster T.L., Liao C.-T. (2014). Interleukin-6 Signaling Drives Fibrosis in Unresolved Inflammation. Immunity.

[98] Balci A., Düz M.E., Vurmaz A., Çilekar Ş., Kaya F. (2023). Comprehensive Biomarker Analysis of Patients with Idiopathic Pulmonary Fibrosis and Interstitial Lung Disease with Healthy Individuals. Eur. Rev. Med. Pharmacol. Sci..

[99] Hur J., Rhee C.K., Choi J.Y., Jo Y.S. (2025). Experimental Modeling of Pulmonary Fibrosis and Combined Emphysema in Mice Using Repeated Doses of Bleomycin and Cigarette Smoke Extract. Pulm. Pharmacol. Ther..

[100] Kadam A.H., Schnitzer J.E. (2024). Highly Calibrated Relationship Between Bleomycin Concentrations and Facets of the Active Phase Fibrosis in Classical Mouse Bleomycin Model. Int. J. Mol. Sci..

[101] Saito F., Tasaka S., Inoue K.-I., Miyamoto K., Nakano Y., Ogawa Y., Yamada W., Shiraishi Y., Hasegawa N., Fujishima S. (2008). Role of Interleukin-6 in Bleomycin-Induced Lung Inflammatory Changes in Mice. Am. J. Respir. Cell Mol. Biol..

[102] Stancil I.T., Michalski J.E., Hennessy C.E., Hatakka K.L., Yang I.V., Kurche J.S., Rincon M., Schwartz D.A. (2022). Interleukin-6-Dependent Epithelial Fluidization Initiates Fibrotic Lung Remodeling. Sci. Transl. Med..

[103] Kobayashi T., Tanaka K., Fujita T., Umezawa H., Amano H., Yoshioka K., Naito Y., Hatano M., Kimura S., Tatsumi K. (2015). Bidirectional Role of IL-6 Signal in Pathogenesis of Lung Fibrosis. Respir. Res..

[104] Pedroza M., Schneider D.J., Karmouty-Quintana H., Coote J., Shaw S., Corrigan R., Molina J.G., Alcorn J.L., Galas D., Gelinas R. (2011). Interleukin-6 Contributes to Inflammation and Remodeling in a Model of Adenosine Mediated Lung Injury. PLoS ONE.

[105] Saito-Fujita T., Iwakawa M., Nakamura E., Nakawatari M., Fujita H., Moritake T., Imai T. (2011). Attenuated Lung Fibrosis in Interleukin 6 Knock-out Mice after C-Ion Irradiation to Lung. J. Radiat. Res..

[106] Ogata T., Yamazaki H., Teshima T., Tsuchiya T., Nishimoto N., Matsuura N. (2012). Anti-IL-6 Receptor Antibody Does Not Ameliorate Radiation Pneumonia in Mice. Exp. Ther. Med..

[107] Zheng B., Keen K.J., Fritzler M.J., Ryerson C.J., Wilcox P., Whalen B.A., Sahin B., Yao I., Dunne J.V. (2023). Circulating Cytokine Levels in Systemic Sclerosis Related Interstitial Lung Disease and Idiopathic Pulmonary Fibrosis. Sci. Rep..

[108] De Lauretis A., Sestini P., Pantelidis P., Hoyles R., Hansell D.M., Goh N.S.L., Zappala C.J., Visca D., Maher T.M., Denton C.P. (2013). Serum Interleukin 6 Is Predictive of Early Functional Decline and Mortality in Interstitial Lung Disease Associated with Systemic Sclerosis. J. Rheumatol..

[109] Ayaub E.A., Dubey A., Imani J., Botelho F., Kolb M.R.J., Richards C.D., Ask K. (2017). Overexpression of OSM and IL-6 Impacts the Polarization of pro-Fibrotic Macrophages and the Development of Bleomycin-Induced Lung Fibrosis. Sci. Rep..

[110] O’Donoghue R.J.J., Knight D.A., Richards C.D., Prêle C.M., Lau H.L., Jarnicki A.G., Jones J., Bozinovski S., Vlahos R., Thiem S. (2012). Genetic Partitioning of Interleukin-6 Signalling in Mice Dissociates Stat3 from Smad3-Mediated Lung Fibrosis. EMBO Mol. Med..

[111] Le T.-T.T., Karmouty-Quintana H., Melicoff E., Le T.-T.T., Weng T., Chen N.-Y., Pedroza M., Zhou Y., Davies J., Philip K. (2014). Blockade of IL-6 Trans Signaling Attenuates Pulmonary Fibrosis. J. Immunol..

[112] O’Reilly S., Ciechomska M., Cant R., van Laar J.M. (2014). Interleukin-6 (IL-6) Trans Signaling Drives a STAT3-Dependent Pathway That Leads to Hyperactive Transforming Growth Factor-β (TGF-β) Signaling Promoting SMAD3 Activation and Fibrosis via Gremlin Protein. J. Biol. Chem..

[113] Wang J.-H., Zhao L., Pan X., Chen N.-N., Chen J., Gong Q.-L., Su F., Yan J., Zhang Y., Zhang S.-H. (2016). Hypoxia-Stimulated Cardiac Fibroblast Production of IL-6 Promotes Myocardial Fibrosis via the TGF-Β1 Signaling Pathway. Lab. Investig..

[114] Chen F., Lyu L., Xing C., Chen Y., Hu S., Wang M., Ai Z. (2025). The Pivotal Role of TGF-β/Smad Pathway in Fibrosis Pathogenesis and Treatment. Front. Oncol..

[115] Wohlfahrt T., Rauber S., Uebe S., Luber M., Soare A., Ekici A., Weber S., Matei A.-E., Chen C.-W., Maier C. (2019). PU.1 Controls Fibroblast Polarization and Tissue Fibrosis. Nature.

[116] Koli K., Myllärniemi M., Vuorinen K., Salmenkivi K., Ryynänen M.J., Kinnula V.L., Keski-Oja J. (2006). Bone Morphogenetic Protein-4 Inhibitor Gremlin Is Overexpressed in Idiopathic Pulmonary Fibrosis. Am. J. Pathol..

[117] McDonough J.E., Ahangari F., Li Q., Jain S., Verleden S.E., Herazo-Maya J., Vukmirovic M., DeIuliis G., Tzouvelekis A., Tanabe N. (2019). Transcriptional Regulatory Model of Fibrosis Progression in the Human Lung. JCI Insight.

[118] Myllärniemi M., Lindholm P., Ryynänen M.J., Kliment C.R., Salmenkivi K., Keski-Oja J., Kinnula V.L., Oury T.D., Koli K. (2008). Gremlin-Mediated Decrease in Bone Morphogenetic Protein Signaling Promotes Pulmonary Fibrosis. Am. J. Respir. Crit. Care Med..

[119] Vyas B., Ishikawa K., Duflo S., Chen X., Thibeault S.L. (2010). Inhibitory Effects of Hepatocyte Growth Factor and Interleukin-6 on Transforming Growth Factor-Beta1 Mediated Vocal Fold Fibroblast-Myofibroblast Differentiation. Ann. Otol. Rhinol. Laryngol..

[120] Ghanemi A., St-Amand J. (2018). Interleukin-6 as a “Metabolic Hormone”. Cytokine.

[121] Wang C., Hua S., Song L. (2023). Ferroptosis in Pulmonary Fibrosis: An Emerging Therapeutic Target. Front. Physiol..

[122] Armitage A.E., Drakesmith H. (2021). TB or Not TB? Soft Pity Opens the Iron Gates. Blood.

[123] Weiss G., Schett G. (2013). Anaemia in Inflammatory Rheumatic Diseases. Nat. Rev. Rheumatol..

[124] Ho T., Nichols M., Nair G., Radford K., Kjarsgaard M., Huang C., Bhalla A., Lavigne N., Mukherjee M., Surette M. (2022). Iron in Airway Macrophages and Infective Exacerbations of Chronic Obstructive Pulmonary Disease. Respir. Res..

[125] Yang Y., Wang Y., Guo L., Gao W., Tang T.-L., Yan M. (2022). Interaction between Macrophages and Ferroptosis. Cell Death Dis..

[126] Yao M., Liu Z., Zhao W., Song S., Huang X., Wang Y. (2025). Ferroptosis in Idiopathic Pulmonary Fibrosis: Mechanisms, Impact, and Therapeutic Opportunities. Front. Immunol..

[127] Agoro R., Taleb M., Quesniaux V.F.J., Mura C. (2018). Cell Iron Status Influences Macrophage Polarization. PLoS ONE.

[128] Ali M.K., Kim R.Y., Brown A.C., Donovan C., Vanka K.S., Mayall J.R., Liu G., Pillar A.L., Jones-Freeman B., Xenaki D. (2020). Critical Role for Iron Accumulation in the Pathogenesis of Fibrotic Lung Disease. J. Pathol..

[129] Zhong H., Belardinelli L., Maa T., Zeng D. (2005). Synergy between A2B Adenosine Receptors and Hypoxia in Activating Human Lung Fibroblasts. Am. J. Respir. Cell Mol. Biol..

[130] Cardoneanu A., Burlui A.M., Macovei L.A., Bratoiu I., Richter P., Rezus E. (2022). Targeting Systemic Sclerosis from Pathogenic Mechanisms to Clinical Manifestations: Why IL-6?. Biomedicines.

[131] Hernandez-Gonzalez F., Prats N., Ramponi V., López-Domínguez J.A., Meyer K., Aguilera M., Muñoz Martín M.I., Martínez D., Agusti A., Faner R. (2023). Human Senescent Fibroblasts Trigger Progressive Lung Fibrosis in Mice. Aging.

[132] Stuart B.D., Lee J.S., Kozlitina J., Noth I., Devine M.S., Glazer C.S., Torres F., Kaza V., Girod C.E., Jones K.D. (2014). Effect of Telomere Length on Survival in Patients with Idiopathic Pulmonary Fibrosis: An Observational Cohort Study with Independent Validation. Lancet Respir. Med..

[133] Yang M.M., Lee S., Neely J., Hinchcliff M., Wolters P.J., Sirota M. (2024). Gene Expression Meta-Analysis Reveals Aging and Cellular Senescence Signatures in Scleroderma-Associated Interstitial Lung Disease. Front. Immunol..

[134] Álvarez D., Cárdenes N., Sellarés J., Bueno M., Corey C., Hanumanthu V.S., Peng Y., D’Cunha H., Sembrat J., Nouraie M. (2017). IPF Lung Fibroblasts Have a Senescent Phenotype. Am. J. Physiol. Lung Cell. Mol. Physiol..

[135] Ritschka B., Storer M., Mas A., Heinzmann F., Ortells M.C., Morton J.P., Sansom O.J., Zender L., Keyes W.M. (2017). The Senescence-Associated Secretory Phenotype Induces Cellular Plasticity and Tissue Regeneration. Genes Dev..

[136] Młynarska E., Kowalik A., Krajewska A., Krupińska N., Marcinkowska W., Motor J., Przybylak A., Tłustochowicz K., Rysz J., Franczyk B. (2025). Inflammaging and Senescence-Driven Extracellular Matrix Remodeling in Age-Associated Cardiovascular Disease. Biomolecules.

[137] Mora A.L., Rojas M., Pardo A., Selman M. (2017). Emerging Therapies for Idiopathic Pulmonary Fibrosis, a Progressive Age-Related Disease. Nat. Rev. Drug Discov..

[138] Schafer M.J., White T.A., Iijima K., Haak A.J., Ligresti G., Atkinson E.J., Oberg A.L., Birch J., Salmonowicz H., Zhu Y. (2017). Cellular Senescence Mediates Fibrotic Pulmonary Disease. Nat. Commun..

[139] Schafer M.J., Haak A.J., Tschumperlin D.J., LeBrasseur N.K. (2018). Targeting Senescent Cells in Fibrosis: Pathology, Paradox, and Practical Considerations. Curr. Rheumatol. Rep..

[140] Tsitoura E., Vasarmidi E., Bibaki E., Trachalaki A., Koutoulaki C., Papastratigakis G., Papadogiorgaki S., Chalepakis G., Tzanakis N., Antoniou K.M. (2019). Accumulation of Damaged Mitochondria in Alveolar Macrophages with Reduced OXPHOS Related Gene Expression in IPF. Respir. Res..

[141] Govaere O., Cockell S., Tiniakos D., Queen R., Younes R., Vacca M., Alexander L., Ravaioli F., Palmer J., Petta S. (2020). Transcriptomic Profiling across the Nonalcoholic Fatty Liver Disease Spectrum Reveals Gene Signatures for Steatohepatitis and Fibrosis. Sci. Transl. Med..

[142] Poch T., Krause J., Casar C., Liwinski T., Glau L., Kaufmann M., Ahrenstorf A.E., Hess L.U., Ziegler A.E., Martrus G. (2021). Single-Cell Atlas of Hepatic T Cells Reveals Expansion of Liver-Resident Naive-like CD4+ T Cells in Primary Sclerosing Cholangitis. J. Hepatol..

[143] Richeldi L., Costabel U., Selman M., Kim D.S., Hansell D.M., Nicholson A.G., Brown K.K., Flaherty K.R., Noble P.W., Raghu G. (2011). Efficacy of a Tyrosine Kinase Inhibitor in Idiopathic Pulmonary Fibrosis. N. Engl. J. Med..

[144] Wollin L., Wex E., Pautsch A., Schnapp G., Hostettler K.E., Stowasser S., Kolb M. (2015). Mode of Action of Nintedanib in the Treatment of Idiopathic Pulmonary Fibrosis. Eur. Respir. J..

[145] Barratt S.L., Creamer A., Hayton C., Chaudhuri N. (2018). Idiopathic Pulmonary Fibrosis (IPF): An Overview. J. Clin. Med..

[146] Zhang W., Yuan L.-L., Li J.-R., Zheng T.-S., Fan L.-H. (2026). Metabolic Reprogramming by Baicalein Disrupts Fibroblast-Macrophage Crosstalk in Idiopathic Pulmonary Fibrosis. Chem. Biol. Interact..

[147] Schumertl T., Lokau J., Garbers C. (2025). IL-6 Signaling in Immunopathology: From Basic Biology to Selective Therapeutic Intervention. ImmunoTargets Ther..

[148] Yokota S., Imagawa T., Mori M., Miyamae T., Aihara Y., Takei S., Iwata N., Umebayashi H., Murata T., Miyoshi M. (2008). Efficacy and Safety of Tocilizumab in Patients with Systemic-Onset Juvenile Idiopathic Arthritis: A Randomised, Double-Blind, Placebo-Controlled, Withdrawal Phase III Trial. Lancet.

[149] Rose-John S., Jenkins B.J., Garbers C., Moll J.M., Scheller J. (2023). Targeting IL-6 Trans-Signalling: Past, Present and Future Prospects. Nat. Rev. Immunol..

[150] Khanna D., Lin C.J.F., Furst D.E., Goldin J., Kim G., Kuwana M., Allanore Y., Matucci-Cerinic M., Distler O., Shima Y. (2020). Tocilizumab in Systemic Sclerosis: A Randomised, Double-Blind, Placebo-Controlled, Phase 3 Trial. Lancet Respir. Med..

[151] Ghazipura M., Macrea M., Herman D., Barnes H., Knight S.L., Silver R.M., Montesi S.B., Raghu G., Hossain T. (2024). Tocilizumab in Patients with Systemic Sclerosis-Associated Interstitial Lung Disease: A Systematic Review and Meta-Analysis. Ann. Am. Thorac. Soc..

[152] Franco G., Le Guen P., Le Brun M., Philippot Q., Dupin C., Debray M.-P., Bancal C., Taillé C., Borie R., Crestani B. (2024). Lack of Efficacy of Tocilizumab in Acute Exacerbation of Pulmonary Fibrosis. ERJ Open Res..

[153] Miles T., Hoyne G.F., Knight D.A., Fear M.W., Mutsaers S.E., Prêle C.M. (2020). The Contribution of Animal Models to Understanding the Role of the Immune System in Human Idiopathic Pulmonary Fibrosis. Clin. Transl. Immunol..

[154] Pianigiani T., Dilroba A., Gambini C., Hernandez-Gonzalez F., d’Alessandro M., Bergantini L., Sellares J., Bargagli E., Cameli P. (2026). Beyond Inflammation: Alarmins as Critical Drivers of Pulmonary Fibrosis. ERJ Open Res..

[155] Namakanova O.A., Gubernatorova E.O., Chicherina N.R., Zvartsev R.V., Drutskaya M.S. (2024). Experimental mouse model of pulmonary fibrosis induced by nebulized LPS administration. Russ. J. Immunol..

[156] Sen’kova A.V., Bishani A., Savin I.A., Zenkova M.A., Chernolovskaya E.L. (2025). Effect of Immunostimulatory RNA on the Fibrosis Development in Bleomycin- or LPS-Induced Mouse Models. Biochimie.

[157] Chicherina N.R., Gorshkova E.A., Myachev E.A., Yakovleva A.S., Litvinova A.M., Namakanova O.A., Zvartsev R.V., Nedospasov S.A., Drutskaya M.S. (2024). A Mouse Model of Overexpression of Human IL-6 by Tissue-Resident Macrophages in the Context of LPS-Induced Inflammation. Med. Immunol..

[158] Koike A., Hayashi K., Fujimori K. (2024). Involvement of Necroptotic Cell Death in Macrophages in Progression of Bleomycin and LPS-Induced Acute Exacerbation of Idiopathic Pulmonary Fibrosis. Eur. J. Pharmacol..

[159] King E.M., Zhao Y., Moore C.M., Steinhart B., Anderson K.C., Vestal B., Moore P.K., McManus S.A., Evans C.M., Mould K.J. (2024). Gpnmb and Spp1 Mark a Conserved Macrophage Injury Response Masking Fibrosis-Specific Programming in the Lung. JCI Insight.

[160] Namakanova O.A., Gorshkova E.A., Zvartsev R.V., Nedospasov S.A., Drutskaya M.S., Gubernatorova E.O. (2022). Therapeutic Potential of Combining IL-6 and TNF Blockade in a Mouse Model of Allergic Asthma. Int. J. Mol. Sci..

[161] Gubernatorova E.O., Namakanova O.A., Gorshkova E.A., Medvedovskaya A.D., Nedospasov S.A., Drutskaya M.S. (2021). Novel Anti-Cytokine Strategies for Prevention and Treatment of Respiratory Allergic Diseases. Front. Immunol..

[162] Lechner A., Henkel F.D.R., Hartung F., Bohnacker S., Alessandrini F., Gubernatorova E.O., Drutskaya M.S., Angioni C., Schreiber Y., Haimerl P. (2022). Macrophages Acquire a TNF-Dependent Inflammatory Memory in Allergic Asthma. J. Allergy Clin. Immunol..

[163] Gubernatorova E.O., Gorshkova E.A., Namakanova O.A., Zvartsev R.V., Hidalgo J., Drutskaya M.S., Tumanov A.V., Nedospasov S.A. (2018). Non-Redundant Functions of IL-6 Produced by Macrophages and Dendritic Cells in Allergic Airway Inflammation. Front. Immunol..

[164] Efimov G.A., Kruglov A.A., Khlopchatnikova Z.V., Rozov F.N., Mokhonov V.V., Rose-John S., Scheller J., Gordon S., Stacey M., Drutskaya M.S. (2016). Cell-Type-Restricted Anti-Cytokine Therapy: TNF Inhibition from One Pathogenic Source. Proc. Natl. Acad. Sci. USA.

